# Genomic-Driven Identification of Conserved Biosynthetic Gene Clusters in *Cladosporium limoniforme*: The Case of the DHN-Melanin Pathway

**DOI:** 10.3390/metabo16010077

**Published:** 2026-01-16

**Authors:** Angela Rojas-Coll, José-Ignacio Valencia, Javier Tognarelli, Guillermo Fernández-Bunster

**Affiliations:** 1 Laboratorio de Biotecnología (BioTecLab), Escuela de Tecnología Médica, Facultad de Medicina, Campus San Felipe, Universidad de Valparaíso, Valparaíso 2100000, Chile; angela.rojasc@estudiantes.uv.cl (A.R.-C.); jose.valenciav@estudiantes.uv.cl (J.-I.V.); 2 Genómica UV, Escuela de Medicina, Facultad de Medicina, Universidad de Valparaíso, Valparaíso 2340000, Chile; javier.tognarelli@uv.cl

**Keywords:** genome mining, secondary metabolites, gene cluster, endolichenic fungi, *Cladosporium limoniforme*

## Abstract

**Background**: Endolichenic fungi represent an emerging source of bioactive secondary metabolites; however, the genomic basis of their chemical diversity remains largely poorly characterized. Specifically, the metabolic capabilities of *Cladosporium limoniforme* have not been explored at the genomic level. **Objectives**: This study aimed to characterize the biosynthetic potential of *C. limoniforme* by presenting its first whole-genome sequence and conducting a comparative analysis of its biosynthetic gene clusters (BGCs), with a specific focus on the evolutionary conservation of the DHN-melanin pathway. **Methods**: Genome mining was performed using antiSMASH and fungiSMASH tools. Comparative genomics involved heatmap-based distribution analysis across the *Cladosporium* genus, synteny profiling using Clinker to assess gene order conservation, and Maximum Likelihood phylogenetic analysis of the polyketide synthase (T1PKS) domain. **Results**: We identified 26 putative BGCs, revealing a largely untapped metabolic repertoire. Comparative analysis demonstrated a high degree of conservation for the metachelin C (siderophore) and 1,3,6,8-tetrahydroxynaphthalene (T4HN) clusters across the genus. Notably, synteny and phylogenetic analyses showed that while *C. limoniforme* retains a conserved, ancestral T1PKS core essential for stress survival, it exhibits a significant reduction in accessory genes compared to plant-pathogenic congeners. **Conclusions**: These findings support a “metabolic streamlining” hypothesis driven by the endolichenic lifestyle, where the fungus retains essential protective machinery while shedding costly accessory genes unnecessary in the buffered lichen niche. This study establishes *C. limoniforme* as a valuable genomic resource for future biotechnological research.

## 1. Introduction

Lichens are symbiotic organisms formed by the association of fungi (mycobionts) with photosynthetic partners, such as green algae and/or cyanobacteria (photobionts) [1]. In this relationship, algal cells synthesize organic nutrients, whereas the fungus provides water, minerals, and physical protection. This mutualistic interaction allows lichens to prosper under extreme ecological conditions [2,3], driving the production of specialized secondary metabolites that are essential for survival and adaptation [4]. These metabolites mediate interactions between microorganisms and their environment [5], and many have demonstrated remarkable medicinal, agricultural, and environmental potentials [6,7,8]. However, the slow growth of lichens in nature and the difficulty in culturing them in vitro have hindered the isolation of these compounds.

Consequently, researchers have focused on faster-growing microorganisms such as endolichenic fungi (ELF) [3,8]. These microfungi are taxonomically and functionally distinct from the dominant mycobionts and reside within the lichen thallus without causing apparent symptoms or producing sporulating structures [9,10]. Since the first report of bioactive compounds derived from ELF in 2007 [11], there has been scientific interest in this area. Studies have identified a wide array of chemical classes, including alkaloids, steroids, terpenoids, quinones, xanthones, peptides, and chromenones [9,12]. These compounds exhibit diverse biological activities, including antimicrobial, antitumor, antiviral, and anti-inflammatory properties [13].

Genes encoding secondary metabolite biosynthesis are typically organized into biosynthetic gene clusters (BGCs) [14]. However, under standard laboratory conditions, many of these clusters remain silent because of the absence of specific ecological triggers [15,16]. Genome mining has emerged as a powerful computational strategy to overcome this limitation. By analyzing gene arrangement, this approach predicts BGCs capable of producing secondary metabolites that are otherwise undetectable using traditional culture-based techniques [17,18]. Complementarily, phylogenetic analysis of BGCs facilitates species classification and reveals patterns of genetic divergence, offering insights into microbial diversity [19,20].

The genus *Cladosporium* (Cladosporiaceae; Capnodiales; Ascomycota) [21] comprises cosmopolitan fungi [22] isolated from diverse substrates, including leaves [21,23], fruits [24,25], soils [26], and lichens [27]. A recent review covering the period from 2000 to 2022 [28] reported over 300 secondary metabolites from this genus, with polyketides constituting approximately 48% of the identified compounds. These metabolites exhibit antibacterial, cytotoxic, antiviral, antifungal, and herbicidal activities, making *Cladosporium* a promising candidate for drug discovery and agricultural applications.

*Cladosporium limoniforme* was first described in 2015 [29], but limited information exists regarding its biology, and its biosynthetic potential remains unexplored. Although the genus is known for bioactive compound synthesis, the specific metabolic landscape of *C. limoniforme* remains unknown. This knowledge gap hinders our understanding of BGC composition, homology to known pathways, and conservation relative to other species. Hence, whole-genome sequencing of *C. limoniforme* would provide a unique opportunity to analyze its BGC repertoire.

As an endolichenic fungus inhabiting a protected yet stressful niche, we hypothesize that *C. limoniforme* harbors evolutionarily conserved BGCs for essential stress-protection metabolites, while exhibiting species-specific structural variations in accessory genes driven by ecological adaptation to the host environment. Therefore, this study aimed to evaluate and compare the biosynthetic potential of *C. limoniforme* with other species of the genus using a genome mining approach to identify conserved secondary metabolite clusters.

## 2. Materials and Methods

### 2.1. Fungal Strain and Culture Conditions

The *C. limoniforme* strain was cultivated on Petri dishes containing Potato Dextrose Agar (PDA) medium (2% potato dextrose, 2% agar) at 25 °C for approximately 7 days. Once fungal growth was established, a sterile scalpel was used to cut a small block of mycelium with agar, which was then transferred into a flask containing 50 mL of PDB (2% potato dextrose powder in distilled water). The culture was maintained on an orbital shaker (100 rpm) at room temperature.

### 2.2. DNA Extraction

Genomic DNA extraction was performed at the Laboratory of Biotechnology, University of Valparaíso, San Felipe (UV-SF), using residual mycelium adhering to the flask walls as the starting material. The biomass was recovered, resuspended in 600 µL of nuclease-free water in a 2 mL Eppendorf tube, and vortexed. The suspension underwent enzymatic digestion with Zymolyase (50 mg/mL, 10 µL) at 37 °C for 1 h, with intermittent vortexing every 20 min. Subsequently, cell lysis was supplemented by adding SDS (10%, 70 µL) and proteinase K (20 mg/mL, 5 µL) and incubated at 65 °C for 15 min.

DNA purification was performed using the CTAB method. Briefly, 100 µL of 5 M NaCl and 100 µL of preheated 2% CTAB/0.7% NaCl solution were added to the lysate and incubated at 65 °C for 10 min to precipitate the DNA. Phase separation was performed by adding 750 µL of chloroform–isopropanol (24:1) and centrifuging at 12,000 rpm for 5 min. The aqueous supernatant was recovered, and DNA was precipitated with 450 µL of isopropanol and centrifuged at maximum speed for 15 min to pellet the DNA. The resulting DNA pellet was washed with cold 70% ethanol, air-dried at room temperature for 24 h, and resuspended in 100 µL nuclease-free water. DNA quality was assessed by electrophoresis, and the samples were stored at 4 °C. The extracted DNA was sent to the Genomics UV Facility (Universidad de Valparaíso) for whole-genome sequencing.

### 2.3. Full Genome Sequencing and Genome Assembly

Whole-genome sequencing was performed at the Genomics UV Facility (Universidad de Valparaíso, Chile). The library was prepared using Native Barcoding Kit 24 (ONT, Oxford, United Kingdom) and sequenced using ONT MinION Mk1b platform with a R10 flowcell. Basecalling was performed using the super-accuracy (SUP) model, and reads were demultiplexed using barcodes. Subsequently, raw reads were merged and quality-filtered using Filtlong (v0.2.1), retaining only reads ≥ 1000 bp and the top 90% based on quality scores.

### 2.4. De Novo Assembly and Quality Assessment

De novo genome assembly was performed using Flye (v2.9.6) with the parameters for ONT high-quality reads (--nano-hq) and an estimated genome size of 34 Mb. The draft assembly underwent polishing through two iterative rounds of Racon (v1.5), followed by a final consensus polishing step using Medaka (v2.0.1) with model r1041_e82_400bps_sup_variant_v5.0.0. Read alignment required for consensus generation was performed using minimap2 (v2.30).

Assembly statistics, including total length, number of contigs, N50, and GC content, were calculated using QUAST (v5.3.0). Genome completeness was assessed using BUSCO (v6.0.0) against the fungi_odb12 dataset on the nuclear assembly and the predicted proteome. Mitochondrial contigs were identified based on elevated sequencing depth and excluded prior to downstream analysis.

### 2.5. Structural and Functional Annotation

Repetitive elements were identified using RepeatModeler (v2.0.7) and soft-masked with RepeatMasker (v4.2.1). Structural gene prediction was performed on the soft-masked nuclear assembly using funannotate predict (v1.8.17), integrating ab initio gene prediction with GeneMark-ES and BUSCO-guided species-specific training with Augustus, assuming diploid ploidy. Functional annotation was performed using funannotate, combining homology-based annotation with DIAMOND (v2.1.10), conserved domain detection using HMMER (v3.4), and orthology assignment using eggNOG-mapper (v2.1.13) with a locally downloaded database.

### 2.6. Analysis of BGCs of Secondary Metabolites from C. limoniforme

The nuclear genome assembly of *C. limoniforme* was mined for BGCs using the Antibiotics and Secondary Metabolite Analysis Shell (antiSMASH v8.0) [30]. The analysis was conducted with the detection strictness set to “relaxed” and all additional features enabled to allow for the preliminary identification of genomic regions potentially associated with secondary metabolism. To refine the predictions specifically for fungal genomes, the sequences were subsequently analyzed using the fungal-dedicated platform fungiSMASH (v8.0) [31] under identical parameter settings.

### 2.7. Selection of Comparative Species

For comparative genomic analyses, eight representative species of *Cladosporium* were selected from the National Center for Biotechnology Information (NCBI) database [32] (Table 1). The selection criteria prioritized genomes designated as “reference assemblies” and aimed to maximize ecological diversity by including isolates from distinct hosts and environments to minimize sampling redundancy.

### 2.8. Prediction of BGCs in Comparative Genomes

BGCs were predicted in all comparative genomes using antiSMASH. Additionally, fungiSMASH was employed for genomes, where structural annotation files (*. gff) were available along with the FASTA sequences. All analyses were performed using the same parameter settings applied to the *C. limoniforme* reference run to ensure methodological consistency across the dataset.

### 2.9. Comparative Profiling and Visualization of BGCs

To evaluate the conservation patterns of the identified BGCs across the *Cladosporium* genus, a comparative profiling analysis was performed between *C. limoniforme* and those identified in the eight selected species. Table S1 lists the genomic coordinates, cluster type, predicted product, similarity to the closest match in the MIBiG database, and source of the predicted cluster for each species in the study. A binary presence/absence matrix was constructed based on the structural annotation and orthology results, where BGCs were scored as present (1) or absent (0) depending on the presence of core biosynthetic enzymes. The resulting dataset was visualized as a heatmap to elucidate lineage-specific distribution patterns and metabolic variations among the analyzed species.

### 2.10. Structural Comparison of BGCs

Gene cluster synteny and structural conservation were analyzed using Clinker (v1.0) [33] and its visualization module clustermap.js. Comparisons included *C. limoniforme*, the selected *Cladosporium* species, and a reference BGC identified using the KnownClusterBlast function of fungiSMASH to provide external context. Selected BGCs were exported in GenBank format (. gbf) and aligned in Clinker to generate similarity matrices based on the sequence identity and gene order. Visualizations were generated using clustermap.js, scaling clusters to size and highlighting homologous genes through identity-coded linkages.

### 2.11. Phylogenetic Analysis of the Core Synthase

To evaluate the evolutionary relationships of the selected central biosynthetic gene, amino acid sequences of the putative T1PKS were extracted from the antiSMASH predictions for *C. limoniforme* and other *Cladosporium* species harboring the T4HN cluster. Sequence alignment was performed using the ClustalW algorithm implemented in MEGA 12 [34], with default parameters. Phylogenetic inference was conducted using the Maximum Likelihood (ML) method based on the best-fit amino acid substitution model determined by the model-selection feature in MEGA. The analysis assumed uniform evolutionary rates among the sites and included all alignment positions. Node support was evaluated using 1000 bootstrap replicates. The phylogenetic tree was rooted using the T1PKS sequence from the *Pestalotiopsis fici* reference cluster, as an outgroup.

## 3. Results

### 3.1. General Features of the Cladosporium limoniforme Genome

The de novo assembly of the *C. limoniforme* nuclear genome yielded a total size of 28.3 Mb, distributed across 20 contigs, with an N50 of 1.66 Mb and GC content of 53.5%. BUSCO analysis (fungi_odb10 dataset) indicated a completeness of 98.6% (Table 2), confirming a high-quality assembly suitable for functional annotation purposes. Gene prediction identified 10,123 protein-coding genes. The mitochondrial genome was assembled into a single circular contig of 27,819 bp with a GC content of 28.5%.

### 3.2. Prediction of Biosynthetic Gene Regions in C. limoniforme

Genome mining using antiSMASH identified 26 genomic regions associated with secondary metabolite biosynthesis in *C. limoniforme*, of which only three showed potential matches with previously reported clusters in the MIBiG database. In comparison, fungiSMASH predicted 21 regions, four of which corresponded to known clusters (Table 3).

Among the clusters showing similarity to previously characterized metabolites, compounds belonging to the NRPS (Non-Ribosomal Peptide Synthetase), T1PKS (Type I Polyketide Synthase), and terpene classes were identified (Table 4). Only two metabolites, 1,3,6,8-tetrahydroxynaphthalene (T4HN) and metachelin C, were consistently predicted by both tools, although fungiSMASH annotated the former as 1,3,8-trihydroxynaphthalene (T3HN). Furthermore, antiSMASH predicted one metabolite that was not detected by fungiSMASH, whereas fungiSMASH identified two metabolites that were absent in the antiSMASH results. Through this combined analysis, five BGCs associated with known metabolites were identified.

### 3.3. Overview of BGCs in Cladosporium Species

All examined species exhibited a limited number of BGCs with homology to known clusters (Table 5). The total repertoire of predicted genomic regions ranged from 22 to 40 across datasets. Among the species analyzed, *C. oxysporum* exhibited the largest biosynthetic potential, whereas *C. cladosporioides* displayed the lowest number of detected clusters.

### 3.4. Comparison of BGC Patterns in the Genus Cladosporium

**Figure 1:**
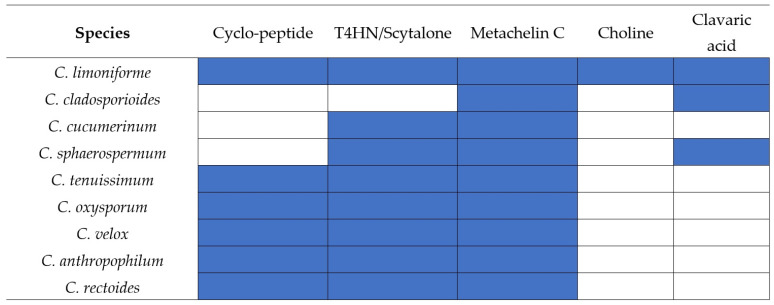
Heatmap of BGCs conservation across the Cladosporium genus. Presence (colored squares) and absence (white squares) of the five main BGCs identified in the *C. limoniforme* genome compared to eight related *Cladosporium* species. The core melanin pathway (T4HN/Scytalone) and Metachelin C are broadly conserved, whereas secondary clusters such as Choline and Clavaric acid show a discontinuous distribution, supporting the hypothesis of lineage-specific gene loss or acquisition.

Comparative profiling of the known BGCs in *C. limoniforme* and related species (Figure 1) revealed lineage-specific conservation patterns. The BGC associated with metachelin C was identified in all analyzed genomes, indicating its ubiquitous distribution within the genus. Similarly, the 1,3,6,8-tetrahydroxynaphthalene (T4HN)/scytalone-T3HN cluster was detected in nearly all species, suggesting that it represents a core metabolic feature of *Cladosporium*. The Cyclo-(D-Phe-L-Phe-D-Val-L-Val) cluster exhibited an intermediate distribution, being present in over half of the species. In contrast, clavaric acid was restricted to only two species, whereas the choline cluster was absent in all comparative genomes. Given the pivotal role of melanin in conferring protection against abiotic stress in lichenized environments, subsequent evolutionary analyses will focus on this specific pathway.

### 3.5. Structural Conservation of the Analyzed BGC

**Figure 2:**
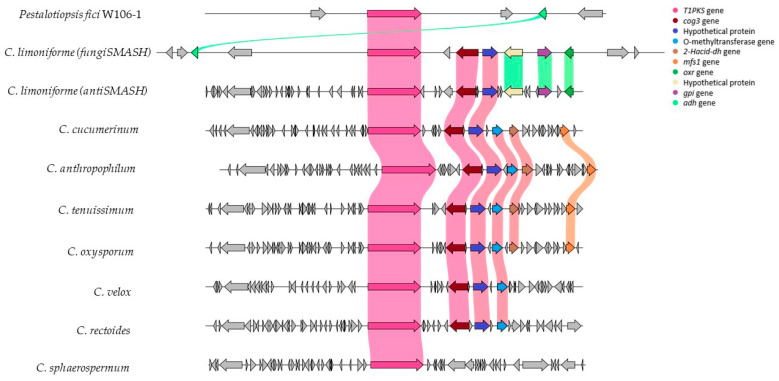
Comparative gene conservation of the repeated BGC T4HN in *Cladosporium* using Clinker. The first cluster corresponded to the reference BGC (*Pestalotiopsis fici*), followed by the fungiSMASH and antiSMASH predictions for *C. limoniforme*. The remaining seven clusters represented other species within the genus *Cladosporium*. A conserved biosynthetic core (pink) was observed, along with variations in accessory genes, reflecting differences in cluster architecture among species. CogE: Component Of Oligomeric Golgi Complex 3, 2-Hacid_dh: 2-hydroxyacid dehydrogenase, MFS1: major facilitator superfamily transporter, gpi: glucose-6-phosphate isomerase, adh: alcohol dehydrogenase.

Comparative synteny analysis using Clinker (Figure 2) demonstrated a high degree of structural conservation between the reference BGC from *Pestalotiopsis fici*, *C. limoniforme*, and other members of the genus. The core region of the BGC, which encodes the biosynthetic T1PKS, was present in all evaluated species. In contrast, greater variability was observed in accessory genes, such as O-methyltransferases and hypothetical proteins. These findings suggest that while the core biosynthetic machinery is evolutionarily conserved, potentially representing the minimal requirement for T4HN expression, structural differences in the flanking regions indicate that species-specific auxiliary components may differentially modulate the biosynthetic pathway.

### 3.6. Evolutionary Relationship of the T1PKS Among Species of the Genus Cladosporium

**Figure 3:**
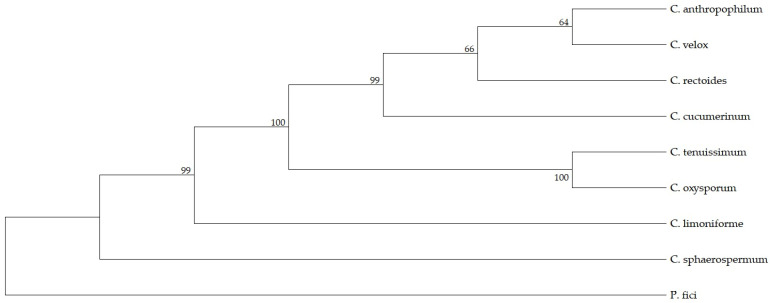
Maximum Likelihood phylogenetic tree of T1PKS amino acid sequences. Bootstrap values are indicated at the nodes. *Pestalotiopsis fici was* used as an outgroup.

Phylogenetic reconstruction (Figure 3) revealed a high degree of sequence conservation among the clustered sequences, indicating that the core T1PKS gene has been evolutionarily preserved within the genus. Notably, the *C. limoniforme* sequence was positioned closer to the basal node (rooted with *P. fici*) than several other analyzed species. This topological placement suggests that this fungus retains the ancestral structural and functional characteristics of T1PKS, driving the T4HN pathway.

## 4. Discussion

This study represents the first comparative assessment of the biosynthetic potential of *C. limoniforme* using two complementary genome mining tools: antiSMASH and fungiSMASH. The analysis predicted 26 and 21 BGCs. Notably, in both datasets, fewer than 20% of the predicted clusters exhibited significant similarity to previously characterized pathways, emphasizing that *C. limoniforme* possesses a vast and largely unexplored metabolic repertoire. Based on homology, five putative BGCs were associated with known metabolic pathways. Specifically, regions 16.2 and 17.2 were consistently detected by both platforms, whereas region 9.3 was unique to antiSMASH and regions 9.1 and 9.2 were exclusively identified by fungiSMASH.

Regarding the specific annotation of the melanin-associated cluster, a divergence in prediction was observed: antiSMASH annotated the product as 1,3,6,8-tetrahydroxynaphthalene (T4HN), whereas fungiSMASH identified it as 1,3,8-trihydroxynaphthalene (T3HN). However, these differences are not considered biologically critical, as both compounds are consecutive intermediates in the same DHN-melanin biosynthetic pathway [35,36]. Such discrepancies in automated predictions have been previously documented [37,38] and are likely attributable to the distinct algorithms, reference databases, and scoring parameters employed by each platform. For instance, a comparative study [39] evaluating specialized metabolite prediction across antiSMASH, fungiSMASH, SMURF, and PRISM demonstrated that while tools may differ in metabolite categorization or similarity scoring, the total count and overall composition of the predicted clusters remain largely consistent. Similar patterns have been reported in eukaryotic algal genomes [40], where tools generally agree on cluster detection but vary in specific metabolite assignment or gene count, reinforcing the necessity of manual curation and multi-tool approaches.

Comparative genomic profiling revealed that two BGCs are highly conserved across the *Cladosporium* genus: the metachelin C cluster, which was present in all analyzed genomes, and the T4HN cluster, which was detected in seven of the eight species. The ubiquitous presence of the metachelin C cluster underscores its importance. Metachelin C is a siderophore responsible for iron acquisition, a critical process for fungal survival, given that the bioavailability of this essential element is often limited by its oxidation into insoluble ferric hydroxides [41]. Beyond its physiological role, this compound has significant biotechnological potential. In agriculture, it functions as a biocontrol agent to enhance plant growth and suppress pathogens; in bioremediation, it facilitates the mobilization of heavy metals and radionuclides; and in the food industry, it is under investigation for its potential as a natural antioxidant [42].

In contrast, the broad conservation of the T4HN cluster highlights the evolutionary value of the DHN-melanin pathway [35]. Melanin is a dark pigment known to confer resistance to a wide range of abiotic stressors, including UV and ionizing radiation, extreme temperatures, and heavy metal toxicity [43]. Consequently, this pathway is of significant interest in biotechnology. In the biomedical field, fungal melanins have demonstrated radioprotective and antioxidant properties, offering protection against oxidative stress and ionizing radiation. Furthermore, in environmental biotechnology, melanin has been proposed as an effective agent for bioremediation because of its physicochemical capacity to adsorb and sequester environmental contaminants, such as heavy metals [43,44]. Melanin plays a pivotal role in survival and stress adaptation. Its dark pigmentation confers resistance to abiotic stressors, such as UV radiation, desiccation, extreme temperatures, and oxidative stress, which are critical for survival within the lichen thallus [45,46,47]. Beyond individual protection, this metabolite likely contributes to the stability of the symbiotic partnership by enhancing the overall health and homeostasis of the lichen ecosystem [3]. Given the high conservation and ecological relevance of the predicted DHN-melanin BGC, a comparative synteny analysis was conducted across the species in which the cluster was detected.

Synteny analysis using Clinker confirmed that the core T1PKS gene is strictly conserved across all analyzed *Cladosporium* species, highlighting its importance. Interestingly, comparison with the *Pestalotiopsis fici* reference cluster revealed significant gene loss; only one accessory gene from the reference was retained in *C. limoniforme*, whereas the others were absent throughout the genus. Furthermore, substantial variability was observed in the accessory gene content of *Cladosporium* species. This genomic heterogeneity implies that while T1PKS constitutes the minimal functional unit for melanin backbone synthesis, the flanking accessory genes likely play non-essential but adaptive modulatory roles tailored to specific physiological needs [48].

The conservation of the core DHN-melanin biosynthetic machinery across the genus highlights its fundamental role in basic stress survival, particularly against UV radiation and oxidative stress. However, the observed variability in accessory genes, specifically the absence of certain transporters and modifying enzymes in *C. limoniforme* compared to plant pathogens such as *C. fulvum*, suggests niche-specific adaptive pressures. We hypothesize that the endolichenic lifestyle of *C. limoniforme* significantly influences its genomic architecture. Unlike environmental fungi, which are directly exposed to fluctuating environmental extremes and host immune responses, *C. limoniforme* inhabits a protected microenvironment within the lichen thallus. This symbiotic niche likely provides physical shielding and chemical homeostasis, potentially reducing the selective pressure to maintain the complex secretion systems or auxiliary enzymes typically required for pathogenicity or host invasion. Consequently, the melanin pathway in *C. limoniforme* appears to reflect an evolutionary ‘optimization’ strategy, maintaining the essential core for cellular protection while shedding metabolically costly accessory functions unnecessary for an endolichenic existence.

Our genome-driven approach used synteny analysis to elucidate the conservation of gene order and content, providing insights into the functional and evolutionary dynamics of the T4HN cluster. The identification of conserved biosynthetic cores among variable accessory regions emphasizes the utility of synteny in revealing taxon-specific gene arrangements that contribute to key adaptive traits [49]. Moreover, this context-based analysis facilitated the functional exploration of annotated “hypothetical proteins” within the cluster, suggesting shared regulatory or synergistic associations with the core synthase [50]. Beyond functional inference, integrating synteny data refines the structural annotation of these loci [51] and enhances the robustness of ortholog identification for phylogenomic reconstruction [52]. Consequently, this comparative framework reinforces the phylogenetic placement of *C. limoniforme* by considering gene order conservation and cluster architecture as complementary evolutionary signals to the sequence data [53].

Phylogenetic reconstruction of the T1PKS domain revealed that while *C. limoniforme* clusters within the melanin-producing lineage of *Cladosporium*, its sequence is topologically closer to the reference gene (*P. fici*) than to those of other congeneric species. This evolutionary proximity suggests that *C. limoniforme* retains a more ancestral variant of T1PKS, consistent with its conserved core structure and lack of extensive accessory insertions. This pattern—a highly conserved core coupled with variable accessory genes—has been documented in other fungal BGCs, such as those for aflatoxin and sterigmatocystin in *Aspergillus*, where peripheral genes modulate catalytic efficiency or intermediates without altering the central biosynthetic module [48].

This structural economy contrasts with the evolutionary patterns observed in obligate parasitic fungi, such as powdery mildew and rust. In these groups, strict host dependence often drives reductive evolution, leading to massive loss of genes involved in independent metabolism and environmental sensing [54,55,56]. Conversely, endolichenic fungi, such as *C. limoniforme*, which live asymptomatically and facultatively within the thallus, generally do not exhibit such drastic genomic erosion, retaining diverse BGCs for environmental interaction [3,13]. However, the specific absence of accessory genes in the T4HN cluster of *C. limoniforme* suggests a nuanced evolutionary scenario: rather than undergoing the generalized reductive evolution typical of parasites, *C. limoniforme* appears to have undergone a targeted “metabolic streamlining.” The lichen host may provide specific precursors or physical protection that renders the full accessory machinery redundant, allowing the fungus to maintain only the essential ancestral core required for melanin synthesis.

To definitively characterize the biosynthetic capability of this BGC, heterologous expression is the necessary next step. Since bioinformatic prediction does not guarantee metabolite production under native conditions, expressing the full cluster or minimal combinations (e.g., T1PKS alone vs. T1PKS plus accessory genes) in model hosts such as *Escherichia coli*, *Saccharomyces cerevisiae*, or *Aspergillus oryzae* would confirm T4HN synthesis [57,58]. Furthermore, to elucidate the specific functions of the annotated “hypothetical proteins” and accessory genes, gene knockout experiments in the native host (if transformable) or comparative heterologous expression could be employed. Following this, chemical profiling via LC-MS and structural elucidation by NMR would reveal whether these accessory genes introduce specific modifications or confer adaptive properties, providing a precise understanding of the functional evolution of BGCs in *C. limoniforme*.

This study has inherent limitations associated with the predictive nature of genome mining. First, tools such as fungiSMASH are continuously evolving; thus, the results are constrained by the software version and algorithms available at the time of analysis. Second, the quality of the genome assembly is critical, as fragmented contigs may artificially split BGCs, leading to incomplete cluster prediction. Discrepancies between tools (e.g., antiSMASH vs. fungiSMASH) regarding ORF identification and hypothetical protein annotation can also introduce uncertainty, whereas the accuracy of metabolic inference relies heavily on the completeness of reference databases such as MIBiG. Therefore, these bioinformatic predictions should be interpreted as guiding hypotheses rather than definitive proofs of metabolism.

Finally, while this study provides robust genomic insights, inherent limitations must be acknowledged beyond computational boundaries. The comparative analysis was constrained by the limited number of high-quality *Cladosporium* genomes currently available from diverse ecological niches, particularly extreme environments. This taxonomic sampling bias may obscure the broader evolutionary patterns specific to extremophilic lineages. Furthermore, our ecological hypotheses regarding the loss of accessory genes in *C. limoniforme* rely on genomic presence/absence data from the genome assembly. Future integrative studies combining transcriptomics (RNA-seq) and proteomics under controlled stress conditions (e.g., UV-B exposure or desiccation) are essential to experimentally validate the expression levels of the identified DHN-melanin clusters and confirm their functional roles in the endolichenic adaptation proposed herein.

## 5. Conclusions

Genomic analysis of *C. limoniforme* provides the first comprehensive characterization of its biosynthetic potential through a comparative approach using antiSMASH and fungiSMASH. This strategy enabled the identification of five putative BGCs associated with the known metabolic pathways. Notably, comparative profiling across the genus revealed that the clusters for metachelin C (a siderophore) and T4HN (a precursor of DHN-melanin) are highly conserved, suggesting that they fulfill essential biological functions related to stress survival and environmental adaptation within *Cladosporium*.

Overall, this study establishes *C. limoniforme* as a valuable genomic resource harboring a largely underexplored yet biologically significant metabolic repertoire. The integration of structural synteny analysis with phylogenetics proved to be a robust strategy for prioritizing clusters of functional interest, offering critical insights into the retention of ancestral biosynthetic features in this species compared to other isolates. These findings lay a solid foundation for future experimental work aimed at functionally validating the T4HN BGC, opening new perspectives for understanding the evolutionary dynamics of secondary metabolism in endolichenic fungi and exploring their potential for biotechnological applications.

## Figures and Tables

**Table 1:** Species of *Cladosporium* considered in this study for the analysis of BGCs.

Species	GenBank	Genome Size	Number of Contigs	Contig N50	GC Percent	Genome Depth	Host/Isolation Source
*C. cladosporioides*	GCA_002901145.1	33.2 Mb	67	2 Mb	52.2	374.29×	Taxus cuspidata (seeds)
*C. cucumerinum*	GCA_023634325.1	33.8 Mb	29	2.1 Mb	51.5	247.27×	Cucumis sativus
*C. sphaerospermum*	GCA_023621355.1	28.1 Mb	436	691.5 kb	55.5	100×	Homo sapiens (feces)
*C. tenuissimum*	GCA_046128905.1	32.7 Mb	33	2.1 Mb	53	170×	Luffa aegyptiaca (leaf)
*C. oxysporum*	GCA_035771495.1	34.5 Mb	52	1.9 Mb	53	300×	Solanum lycopersicum
*C. velox*	GCA_024604135.1	32 Mb	21	1.7 Mb	53	154×	Gossypium sp. (cotton field)
*C. anthropophilum*	GCA_052324185.1	30.8 Mb	83	1.2 Mb	53	270×	Citrus x limon
*C. rectoides*	GCA_046128805.1	31.4 Mb	19	2.1 Mb	52.5	180×	Soil at roots of Citrus reticulata

**Table 2:** Genome assembly statistics and completeness of *C. limoniforme*.

Metric	Value
Genome size (Mb)	28.3
Number of contigs	20
Genome depth	28.9×
N50 (Mb)	1.66
GC content (%)	53.5
BUSCO completeness (%)	98.6
BUSCO single-copy (%)	98.5
BUSCO duplicated (%)	0.1
BUSCO fragmented (%)	0.1
BUSCO missing (%)	1.2

**Table 3:** Results from antiSMASH and fungiSMASH for *C. limoniforme*.

Tool	Total BGCs	Known BGCs	Unknown BGCs
antiSMASH	26	3	23
fungiSMASH	21	4	17

**Table 4:** Known BGCs identified in *C. limoniforme* using antiSMASH and fungiSMASH.

Tool	Region	Cluster Type	Similar Product (MiBiG)
antiSMASH	9.3	NRPS	Cyclo-(D-Phe-L-Phe-D-Val-L-Val)
antiSMASH	16.2	T1PKS	1,3,6,8-tetrahydroxynaphthalene
antiSMASH	17.2	NRPS	metachelin C
fungiSMASH	9.1	NRPS-like	choline
fungiSMASH	9.2	Terpene	clavaric acid
fungiSMASH	16.2	T1PKS	scytalone/T3HN
fungiSMASH	17.2	NRPS	metachelin C

**Table 5:** Known and unknown BGCs identified in the *Cladosporium* genomes.

Species	Total BGCs	Known BGCs	Unknown BGCs
*C. cladosporioides*	22	3	19
*C. cucumerinum*	27	4	23
*C. sphaerospermum*	29	7	22
*C. tenuissimum*	36	6	30
*C. oxysporum*	40	9	31
*C. velox*	31	5	26
*C. anthropophilum*	34	6	28
*C. rectoides*	34	6	28

## Data Availability

The genome assembly of Cladosporium limoniforme is available at DDBJ/ENA/GenBank under accession JBTNQL000000000 (version JBTNQL010000000), associated with BioProject PRJNA1398797 and BioSample SAMN54460825.

## Supplementary Materials

The following supporting information can be downloaded at: https://www.mdpi.com/article/10.3390/metabo16010077/s1, Table S1. Detailed list of putative biosynthetic gene clusters (BGCs) showing similarity to known pathways identified in the *Cladosporium* reference species using antiSMASH.
