# Supplementary material for: Genomic-Driven Identification of Conserved Biosynthetic Gene Clusters in Cladosporium limoniforme: The Case of the DHN-Melanin Pathway
Source: Metabolites. 2026 Jan 16;16(1):77. doi: 10.3390/metabo16010077 (PMC12843665; doi:10.3390/metabo16010077)
Supplement: Supplementary file 1 [file metabolites-16-00077-s001.zip › Table S1.docx]

**Table S1.** Detailed list of putative biosynthetic gene clusters (BGCs) showing similarity to known pathways identified in the *Cladosporium* reference species using antiSMASH.

| Species | Region | Cluster type | Similar product (MiBiG) | Similarity | BGC source (organism) |
| --- | --- | --- | --- | --- | --- |
| *C. cladosporioides* | 7,4 | NRPS | metachelin C | Low | *Metarhizium robertsii ARSEF 23* |
|  | 63,1 | NRPS | nidulanin A | Low | *Aspergillus nidulans FGSC A4* |
|  | 63,3 | terpene | clavaric acid | High | *Clavariadelphus truncatus* |
| *C. cucumerinum* | 9,3 | NRPS | nidulanin A | Low | *Aspergillus nidulans FGSC A4* |
|  | 11,3 | NRPS, fungal-RiPP | metachelin C | Low | *Metarhizium robertsii ARSEF 23* |
|  | 11,4 | T1PKS | cercosporin | Low | *Cercospora beticola* |
|  | 14,2 | T1PKS | 1,3,6,8-tetrahydroxynaphthalene | High | *Glarea lozoyensis* |
| *C. sphaerospermum* | 31,1 | T1PKS | fusarin C | High | *Fusarium fujikuroi* |
|  | 64,1 | T1PKS | fusarin C | High | *Fusarium fujikuroi* |
|  | 112,2 | NRPS-like | nidulanin A | Low | *Aspergillus nidulans FGSC A4* |
|  | 112,5 | terpene | clavaric acid | High | *Clavariadelphus truncatus* |
|  | 238,1 | NRPS-like, terpene | aspyridone A | Low | *Aspergillus nidulans FGSC A4* |
|  | 291,2 | NRPS | metachelin C | Low | *Metarhizium robertsii ARSEF 23* |
|  | 302,2 | T1PKS | 1,3,6,8-tetrahydroxynaphthalene | High | *Glarea lozoyensis* |
| *C. tenuissimum* | 2,4 | T1PKS | ajudazol A | Low | *Chondromyces crocatus* |
|  | 4,3 | T1PKS | compactin | Low | *Penicillium citrinum* |
|  | 8,5 | NRPS | cyclo-(D-Phe-L-Phe-D-Val-L-Val) | High | *Penicillium rubens Wisconsin 54-1255* |
|  | 12,1 | T1PKS | 1,3,6,8-tetrahydroxynaphthalene | High | *Glarea lozoyensis* |
|  | 12,2 | T1PKS | Secalonic acid B | Low | *Claviceps purpurea 20.1* |
|  | 19,1 | NRPS, fungal-RiPP | metachelin C | Low | *Metarhizium robertsii ARSEF 23* |
| *C. oxysporum* | 5,1 | T1PKS | compactin | Low | *Penicillium citrinum* |
|  | 14,5 | NRPS | cyclo-(D-Phe-L-Phe-D-Val-L-Val) | High | *Penicillium rubens Wisconsin 54-1255* |
|  | 18,1 | T1PKS | cichorine | High | *Aspergillus nidulans FGSC A4* |
|  | 18,2 | terpene, T1PKS | prolipyrone B | High | *Fusarium graminearum PH-1* |
|  | 20,1 | betalactone | mycosubtilin | Low | *Bacillus subtilis subsp. spizizenii ATCC 6633* |
|  | 20,2 | T1PKS | neosartorin | Low | *Aspergillus novofumigatus IBT 16806* |
|  | 20,3 | T1PKS | 1,3,6,8-tetrahydroxynaphthalene | High | *Glarea lozoyensis* |
|  | 22,1 | NRPS, fungal-RiPP | metachelin C | Low | *Metarhizium robertsii ARSEF 23* |
|  | 51,1 | T1PKS | prolipyrone B | High | *Fusarium graminearum PH-1* |
| *C. velox* | 8,1 | terpene | PR-toxin | Low | *Penicillium roqueforti FM164* |
|  | 10,1 | T1PKS | cercosporin | Low | *Cercospora beticola* |
|  | 11,1 | T1PKS | 1,3,6,8-tetrahydroxynaphthalene | High | *Glarea lozoyensis* |
|  | 14,4 | NRPS | nidulanin A | Low | *Aspergillus nidulans FGSC A4* |
|  | 18,1 | NRPS, fungal-RiPP | metachelin C | Low | *Metarhizium robertsii ARSEF 23* |
| *C. anthropophilum* | 3,1 | NRPS-like, NRPS | ACT-Toxin II | High | *Alternaria alternata* |
|  | 5,1 | NRPS, fungal-RiPP | metachelin C | Low | *Metarhizium robertsii ARSEF 23* |
|  | 6,2 | terpene | PR-toxin | Low | *Penicillium roqueforti FM164* |
|  | 9,2 | T1PKS | 4-epi-15-epi-brefeldin A | Low | *Penicillium brefeldianum* |
|  | 11,4 | NRPS | cyclo-(D-Phe-L-Phe-D-Val-L-Val) | High | *Penicillium rubens Wisconsin 54-1255* |
|  | 16,1 | T1PKS | 1,3,6,8-tetrahydroxynaphthalene | High | *Glarea lozoyensis* |
| *C. rectoides* | 8,5 | NRPS | nidulanin A | Low | *Aspergillus nidulans FGSC A4* |
|  | 11,1 | T1PKS | 1,3,6,8-tetrahydroxynaphthalene | High | *Glarea lozoyensis* |
|  | 11,4 | T1PKS | secalonic acid B | Low | *Claviceps purpurea 20.1* |
|  | 12,1 | T1PKS | alternapyrone | Low | *Alternaria solani* |
|  | 13,1 | NRPS, fungal-RiPP | metachelin C | Low | *Metarhizium robertsii ARSEF 23* |
